# Nurses’ Moral Sensitivity Regarding the Terminally Ill

**DOI:** 10.17533/udea.iee.v37n3e07

**Published:** 2019-10-23

**Authors:** Yolima Carmona González, Amparo Montalvo Prieto

**Affiliations:** 1 Nurse, Masters. Professor, Universidad de Cartagena, Colombia. Email: ycarmonag1@unicartagena.edu.co Universidad de Cartagena Universidad de Cartagena Colombia ycarmonag1@unicartagena.edu.co; 2 Nurse, Masters. Professor, Universidad de Cartagena, Colombia. Email: amontalvop1@unicartagena.edu.co Universidad de Cartagena Universidad de Cartagena Colombia amontalvop1@unicartagena.edu.co

**Keywords:** nursing care, terminally ill, intensive care units, hospitalization, morals, ethics, nursing, surveys and questionnaires, cross-sectional studies., cuidados de enfermagem, doente terminal, unidades de terapia intensiva, hospitalização, princípios morais, ética em enfermagem, inquéritos e questionário, estudos transversais.

## Abstract

**Objective.:**

The purpose, herein, was to determine the moral sensitivity of nurses when caring for terminally ill patients.

**Methods.:**

Descriptive study conducted in the city of Cartagena (Colombia) with the participation of 118 nurses with minimum experience of six months in caring for the terminally ill in general hospitalization, caring for chronic patients, and intensive care units. The study used the 23-item questionnaire on *Moral Sensitivity in Nursing Care - (Sensibilidad Moral en el Cuidado Enfermero* -CuSMCE-23, in spanish) *-* by Campillo, which has six Likert-type response options (0 = total disagreement, to 5 = total agreement) and which has two dimensions: *Nurse values* (12 items) and *Care responses (*11 items*).* A higher score meant a higher degree of moral sensitivity.

**Results.:**

It was found that 89.8% of the participants were women; 20.3% had a graduate degree; 39.8% had less than five years of care experience; 58.5% worked in a public institution - by type of service: 58.5% worked in general hospitalization; 32.2% in the intensive care unit; and 9.3% with chronic patients. The global moral sensitivity regarding the terminally ill in the study group was at 80%. By dimensions, while the *Values* dimension obtained 90%, the *Care responses* dimension only reached 70.4%, with the latter dimension showing difficulties in the items: ‘Often, when I am with a patient, I talk about myself to be more comfortable’ (27.1%), ‘It is hard for me to accept certain decisions by the patients’ (55.1%), and ‘It is hard for me to identify concerns regarding the religious expression’ (60.2%).

**Conclusion.:**

Although the global levels of nurse’s moral sensitivity regarding the terminally ill and of the dimension *Nurse Values* are high, the dimension of *Care responses* has limitations, especially in accepting the diversity of expressions presented by patients.

## Resumo

**Objetivo.:**

Determinar a sensibilidade moral das enfermeiras ao cuidar pacientes terminais.

**Métodos.:**

Estudo descritivo realizado na cidade de Cartagena (Colômbia) com a participação de 118 enfermeiras com experiência mínima de seis meses em cuidado ao doente terminal em hospitalização geral, cuidado ao paciente crônico e unidades de tratamentos intensivos. Se utilizou o Questionário de 23 itens *Sensibilidade Moral no Cuidado Enfermeiro* (CuSMCE-23) de Campillo, o qual tem seis opções de resposta tipo Likert (0 = desacordo total a 5 = acordo total) e duas dimensões: *Valores enfermeiros* (12 itens) e *Respostas de cuidado (*11 itens*).* A maior pontuação maior grau de sensibilidade moral.

**Resultados.:**

Se encontrou que 89.8% eram mulheres, 20.3% havia realizado uma pós-graduação, 39.8% tinha menos de 5 anos de experiência assistencial, 58.5% trabalhava numa instituição pública, por tipo de serviço: 58.5% trabalhava em hospitalização geral, 32.2% na Unidade de Tratamentos Intensivos e 9.3% em pacientes crônicos. A sensibilidade moral global frente ao doente terminal no grupo estudado foi de 80%. Por dimensões, enquanto que na dimensão *Valores* se obteve um 90%, na de *Respostas de cuidado* somente se alcançou 70.4%, tendo esta última dimensão dificuldades nos itens: ‘Com frequência quando estou com o paciente falo de mim mesma para estar mais cômoda’ (27.1%), ‘É difícil aceitar determinadas decisões dos pacientes’ (55.1%) e ‘É difícil identificar inquietudes ao respeito à expressão religiosa’ (60.2%).

**Conclusão.:**

Embora o nível global de sensibilidade moral da enfermeira frente ao doente terminal e da dimensão *Valores enfermeiros* são altos, a dimensão *Respostas de cuidado* apresenta limitações, especialmente na aceitação da diversidade de expressões que apresentam os pacientes.

## Introduction

Human existence imposes the condition of finitude and with it disease and death may appear at any moment of life. In this sense, in Colombia, Legislation 1733 defines as terminally ill the “person with a disease of irreversible and progressive nature, not susceptible to curative treatment and of proven effectiveness, which permits modifying the prognosis of near death; or when the therapeutic resources used with curative purposes have ceased to be effective”.(1)

Cases of patients with chronic noncommunicable diseases (CNCD) and terminally ill patients have increased in all regions of the world due to causes as diverse as increased life expectancy - which surpasses 80 years,(2) - tobacco use, sedentary lifestyle, harmful use of alcohol, and unhealthy diets.(3) In 2016, CNCD caused 71% of deaths in the world (40.5-million), 44% of which were due to cardiovascular disease, 22% due to cancer, 9% caused by respiratory diseases, and 4% due to diabetes.(4) Throughout the world, in 2017, 940 000 people died due to diseases related with HIV.(5) In Colombia, between 2005 and 2014, the principal causes of death in the general population were diseases of the circulatory system in 30.0% and neoplasm that caused 17.9 % of all deaths, with prevalence in adults between 27 and 59 years of age (71.2%) and people over 60 years of age (82.1%).(6) In the department of Bolívar, in 2014, the highest rate of mortality due to CNCD occurred due to ischemic heart disease, with 70.99 deaths in men and 45.47 deaths in women for every 100 000 inhabitants.(7)

The burden represented for the healthcare sector by said diseases in terminal stage increases the world’s need for palliative care at global scale. Currently, it is estimated that 20.4-million people need these cares; 69% of them are adults over 60 years of age and 52% are of male gender. Europe concentrates the highest rates globally: approximately, for every 100 000 inhabitants, between 307.17 and 467.52 people require these cares, followed immediately by countries from the western Pacific region with between 281.64 and 307.16 people and those from the region of the Americas with between 272.66 and 281.64 people. The need for palliative care is concentrated at 38.4% in patients with cardiovascular disease, 34.0% in patients with cancer, and the rest due to pathologies, like HIV/AIDS, diabetes, and Alzheimer’s, among other CNCD.(8) In terminal stage, the person endures numerous and diverse multifactor symptoms, which are changing and, often, cause the loss of their autonomy and quality of life, leading them to the maximum of their vulnerability. The care provided during this phase of the disease is no longer focused on the recovery of health, but on achieving the best quality of life possible for the patients and their families(8) and facilitating the construction of experiences that lead to a serene end of life.(9) 

Regarding the terminally ill, nurses must have the moral sensitivity that allow them to be intuitive, perceiving and being alert to their needs to respond physically, emotionally, and professionally with moral attitudes and behaviors, like empathy, comprehension, and self-awareness,(10) which help them to understand the fragility of the people and to be aware of the implications of the moral decisions made in favor of the patient.(11) In nursing, moral sensitivity permits a nurse-patient relationship centered on trust and the availability to respond to individual needs, which enables or limits the patient’s autonomy to protect them in their vulnerability.(11) A low degree of moral sensitivity may cause nurses to have difficulties in distinguishing between a situation of moral nature from another that is not, that the moral component goes unnoticed or the absence of adequate decision making in favor of the patient; above all, when patients do not accept their disease or the treatment.(12) In this sense, moral sensitivity stands out as an essential aspect to care and recognize dignity to the individual at the end of life, especially when said person has the autonomy to give up unnecessary medical treatments, without therapeutic proportionality, and which do not represent a dignified life.(1)

Factors determining moral sensitivity include gender, age, professional experience, and type of clinical practice.(13) Regarding gender, Lutzen(14) finds that women show greater subjectivity in their moral reasoning, prioritize the interpersonal relationship, individualize and particularize care, expressing greater moral sensitivity through actions that seek protection of the patient’s vulnerability and decision making under the principle of integrality. Men manifest moral sensitivity with greater objectivity: assume moral actions from the obligatory, conceiving autonomy and rules in decision making as priority.(14) Perception, as condition present in moral sensitivity, permits nursing professionals -men or women- to give meaning to their intuition and observation of the care experience from a particular perspective of their values and beliefs; helping them to comprehend the moral dilemmas generated around the patient, as well as discerning about the decisions that must be made and their consequences.(11)

In relation with the perception of caring for the terminally ill, Souza *et al.*(15) describe that nurses participating in their study observed that terminally ill patients endured death with much pain when extraordinary measures were used in treating patients who were not recoverable, with indiscriminate use of advanced therapies that only prolongs the dying process. Bedregal and Zúñiga(16) found that the fear of death impacts upon the moral sensitivity, increasing uncertainty, anxiety, and the perception of this event as a dilemma, which hinders decision making. The purpose of caring for the terminally ill is to propitiate that the cease of biological life does not mean pain and despair, but for it to have a spiritual transcendence for patients and their families. From these considerations, the aim of this study was to determine the moral sensitivity of nurses who care for terminally ill patients.

## Methodology

This was a quantitative study with descriptive design, with the participation of 118 nursing professionals from the services of general hospitalization, care for chronic patients, and intensive care and with at least six months of experience in caring for the terminally ill. The study was conducted in Cartagena (Colombia) during 2017, in five health institutions, one public and four private (two in tier II of care and three in tier III of care, which authorized through written communication the collection of the information. To select the participating institutions, the study kept in mind the hospital centers concentrating the highest number of terminally ill patients. Two research aides participated in collecting the information after receiving training on aspects inherent to this process. The research was estimated without risk;(18) nevertheless, the nurses working in the institutions selected were explained the study objectives and the possibility of withdrawing their participation if they so desired. Thereafter, they signed the informed consent and proceeded to fill out the sociodemographic survey and the Questionnaire on Moral Sensitivity in Nursing Care *(Sensibilidad Moral en el Cuidado Enfermero* -CuSMCE-23, in spanish).(17) 

CuSMCE has 23 statements that evaluate the moral sensitivity of nurses in the dimensions of “values” (12 items) and “care responses” (11 items), with an internal consistency of 0.83 and Pearson’s r coefficient of 0.86.(17) In this study, the Cronbach’s alpha obtained was 0.77 and by dimensions it was 0.78 for values and 0.70 for care responses. Each item was evaluated in a Likert-type scale with six response options, where zero corresponds to “total disagreement”, one means “considerable disagreement”, two is “slight disagreement”, three denotes “slight agreement”, four is “considerable agreement”, and five corresponds to “total agreement”.(17) The data were grouped so that the response options “considerable agreement”, “slight agreement”, and “total agreement” are represented by the term “agree” and the options “total disagreement”, “slight disagreement”, and “considerable disagreement” are identified with the term “disagree”. The data collected were analyzed in the SPSS statistical package, version 21.0. The global score of values of the instrument ranges between a minimum score of 0 and a maximum score of 115, for the dimension of values between 0 and 60 and the dimension of care responses between 0 and 55. Bearing in mind that the score exceeds 50% of the maximum score, it was considered that scores above 58 points indicate high degree of moral sensitivity; above 31 is interpreted as high degree for the dimension of values and for care responses a score >28.

## Results

From the general characteristics of the 118 participating nurses, 89.8% were women; 85.6% were between 20 and 40 years of age; two in every 10 received graduate formation; six in every 10 had over five years in nursing care; and 58.5% work public hospitals in general hospitalization wards (Table 1).

**Table 1:** Sociodemographic characteristics of 118 nurses caring for the terminally ill

Characteristics	Frequency	%
Sex		
Female	106	89.8
Male	12	10.2
Age in years		
20-30	52	44.1
31-40	49	41.5
41 and more	17	14.4
Level of formation		
Undergraduate	94	79.7
Specialist	24	20.3
Work experience in years		
0-5	47	39.8
6-10	35	29.7
11-15	17	14.41
16-20	5	4.24
20 and more	14	11.86
Work area		
Chronics	11	9.3
Hospitalization	69	58.5
ICU	38	32.2
Institution		
Private	49	41.5
Public	69	58.5

The average moral sensitivity found in nurses participating in the study when confronted with a terminal patient was of 92±8.6 points (minimum = 73 and maximum = 110). Regarding the dimensions, in *Values* an average was found of 54±4.8 points (minimum = 41 and maximum = 60) and in *Care responses* an average was obtained of 38.7±6.1 points (minimum = 24 and maximum = 52). The global score obtained by the participants for moral sensitivity was of 80.0%; for the items contained in the dimension of *Values*, it was of 90.0% and for the *Care responses* it was of 70.4% (Table 2).

Upon evaluating the dimension of *Values*, it was found that 100% of the male and female nurses, regarding the terminally ill, agree that in the work setting, it is fundamental to show an attitude of support, establish a relationship of trust, help them to express their concerns to the physician, to realize that they are unique beings, to being attentive to the patients’ expressions to perceive their needs and support them in times of suffering. Between 90% and 99.2% manifested agreement in expressing to the patient their availability as professionals, having the ability to perceive that which worries the patient, demonstrating sincere interest for the patient, and trying to reach a nurse-patient relationship based on honesty. In relation to having time to sit by the patients and listen to them, and feeling bothered when hearing that patients are referred to by their diagnosis had a value <86% (Table 2). 

With respect to the dimension of *Care responses*, it was found that in front of the terminal patient, more than 84% of the participants stated agreement in having to address the person they are caring for in a calm and unhurried voice, demonstrating special interest in proving them comfort, helping them to identify their strengths and capabilities, and debating the patients’ care concerns directly with the physicians implied. Between 60% and 72.0% of the nurses in the study considered that while they are caring for a patient, their head is someplace else; they believe that sometimes they impose their values; they minimize patients’ feelings to avoid their being stressed; they sense difficulty to being available to listen to their feeling; they sense difficulty to identify concerns with respect to religious issues; and often when they are with the patient, they talk about themselves to feel more comfortable; for an average of 55.1% of the participants it is difficult to accept certain decisions by the patients (Table 2).

**Table 2:** Proportion of nurses caring for terminally ill patients who agree with the statements in the QuMSNC-23 scale (*n* = 118)

Dimension / items	Percentage
Dimension of Nurse Values	
For me, it is important as a nurse to express to the patient my availability as professional	99.2
In my work setting, I consider it fundamental to show the patient an attitude of support	100
In my work setting, I consider it fundamental to establish a relationship of trust with the patient	100
I believe that as a nurse, I must help patients express their concerns to the physician	100
I have a special interest in helping patients realize that each being is unique	100
I have the ability to perceive what worries the patient	98.4
I feel that I must assure patients that as caregiver I will be available to support them in times of suffering	100
Being attentive to the patients’ expressions helps me to perceive their needs.	100
It bothers me to hear patients being referred to by their diagnosis.	85.7
It worries me not to have time to sit by the patients and listen to them	88.4
I feel I must show sincere interest for the patient	97.5
I need my relationship with patients to be based on honesty	97.5
*Dimension subtotal*	90.0
Dimension of Care Responses	
It is difficult for me to be willing to listen to the patient’s feelings	67.8
It is hard for me to identify concerns regarding the religious expression	60.2
As a nurse, I think it is not my place to debate the patient’s care concerns directly with the physicians implied	84.7
Sometimes, I believe I impose my values on patients	74.6
Often, when I am with a patient, I talk about myself to be more comfortable	27.1
I try to address patients in a calm and unhurried voice	96.6
Sometimes, I feel I must minimize the patients’ feelings to avoid their being stressed	67.9
I think it is important to help patients to identify their strengths and capabilities	89
I have a special interest in providing comfort to the patients	95.8
Sometimes, I am caring for a patient, but feel my head is someplace else	78
It is hard for me to accept certain decisions by the patients	55.1
*Dimension subtotal*	70.4
*Scale Total*	80.0

Table 3 shows the total average and averages by dimensions of the CuSMCE-23 were only significantly different in the dimension of *Care responses* by type of institution, where the private institution had a higher score than the public*.* Generally, it may be stated that the moral sensitivity score was higher in women, increases with the person’s age, is higher in nurses who only have an undergraduate degree, improves with years of work experience; by type of services, it is superior in chronic care, and by type of institution, the score is higher in private institutions.

**Table 3:** Average of the total score and by domains of the CuSMCE-23 scale according to variables of interest (*n*=118)

		Dimension	Dimension
Characteristics	Total		
		Values	Care Responses
Total	92.7±8.6	54.0±4.8	38.7±6.0
Sex			
Male	91.4±11.1	52.5±6.1	38.9±6.5
Female	92.8±8.3	54.2±4.6	38.6±6.0
p value	0.677	0.362	0.879
Age in years			
20-30	91.3±7.6	53.3±4.5	38.1±5.4
31-40	93.2±9.4	54.2±4.8	39.0±6.4
41 and more	95.3±8.9	55.9±4.9	39.4±6.8
p value	0.224	0.121	0.665
Level of formation			
Specialist	91.8±9.2	52.6±5.3	39.3±6.2
Undergraduate	92.9±8.5	54.4±4.6	38.5±6.0
p value	0.602	0.128	0.576
Work experience in years			
0-5	91.3±8.2	53.2±4.7	38.1±5.3
6-10	92.8±8.2	53.6±4.6	39.2±5.7
11-15	95.2±9.2	55.9±4.2	39.3±7.1
16 and more	93.6±9.7	55.2±5.4	38.4±7.2
p value	0.425	0.150	0.821
Work area			
General hospitalization	91.6±8.7	53.9±5.1	37.69±5.9
ICU	93.9±8.1	54.2±4.4	39.7±6.1
Chronic care	95.4±9.1	54.0±5.0	41.5±4.8
p value	0.219	0.955	0.57
Institution			
Public	91.5±8.9	53.9±5.1	37.6±6.0
Private	94.3±8.0	54.2±4.4	40.1±5.7
p value	0.079	0.691	0.028

## Discussion

The moral sensitivity determined in the nurses participating in the study was of 92.7±8.6 points and corresponds to 80% of the study participants. In this regard, Tas Arslan,(19) in a study on moral sensitivity conducted with pediatric nurses, found a total median score of 95.89 ± 24.34, which determined that over half of the nurses consider themselves competent to recognize and solve ethical problems, supported on their own knowledge or on the participation from the members of the health staff. Dalla(12) found that primary-care nurses have moderate moral sensitivity and express it in the dimensions “interpersonal orientation”, which centers on the construction of a relationship of trust with the patient, and on “professional knowledge” as base to elaborate ethical judgment in care. This author describes, additionally, that conflict and moral significance are the dimensions that denote lower level of moral sensitivity, reflected on the difficulty nurses have in experiencing moral conflicts, understanding them, and assigning them moral content.

With relation to the moral sensitivity and with the population studied not having the maximum degree of moral sensitivity (100%), it is necessary to reflect on which aspects influence positively or negatively on its presence. Park and Kjervik(20) showed in their study that moral sensitivity increases with ethical education, with higher-semester nursing students registering higher scores compared with first-year students. Thus, they conclude that if there is greater formation, reflection, and discussion around ethical themes in the students and the alumni professionals, an increase could be noted in their level of moral sensitivity in the care relations.

As in other studies,(12,13,17,21) the participants were mostly women and although no statistically significant difference was evidenced, they had higher results in total moral sensitivity and in the dimension of values, which is relevant to highlight because being a woman could be a determining factor in moral sensitivity, given that Lutzen(14) indicates a difference between the female and male genders in the way of establishing the physician-nurse-patient relationship and making decisions based on medical and nursing knowledge. Tas Arslan(19) reports that participating female nurses had holistic approaches and a higher moral sensitivity compared with male nurses. Gilligan,(22) in the theory of the ethics of care, expresses that the moral reasoning between men and women is different, arguing that men, generally, bear in mind impartiality I the moral action guided by judgment towards the just, the unjust, and the obligatory. Women, instead, tend to do it from the sphere of the particularity and protection of human fragility. Campillo.(17) differs from the idea expressed previously, given that her research studied the relation between moral sensitivity and gender and reached the conclusion that men and women respond equally when asked about their moral sensitivity in nursing care.

The participants in this study are mostly young adults between 20 and 40 years of age with professional experience under 10 years. In this respect, Lutzen,(11) in relation to age and professional experience, indicates that moral sensitivity increases with age, is developed by experiences, and is expressed differently in groups, that is, each person can conceive differently the importance of the relationship with the patient, respect to autonomy, and becoming aware of the moral consequences. Sayers,(23) however, considers that the biological age does not determine a professional’s level of sensitivity or insensitivity, as do their life experiences and the development of their knowledge, which allow them to create moral conscience of themselves and of the rest. According to said study, nurses were unanimous when expressing that one became a “sensitive being” with experience and as their practice advanced. These findings lead to thinking that female nurses and young male nurses, with less experience in the professional exercise, could have difficulties distinguishing problems of moral nature from those that are not, as well as making ethical decisions when offering care, above all in the terminal disease context.

In this study, the score de moral sensitivity in nurses working in a private institution and in chronic care units was higher than that of those working in a public institution and in general hospitalization. The study by Molouk(24) reports ethical conflict when the institutional values that are part of the work environment are related with the nurses’ values, Dalla(12,13) expresses the need to reflect about the organization, its sustainability, and conformation, given that nurses often make clinical decisions bearing in mind their personal principles and values, but these can lead to conflicts with the staff, the directors, and families of patients. The concern nurses show about the *personal* and *organizational* norms, expresses high ethical sensitivity in the safety and care of patients, and the relationships among coworkers. Within the setting of nursing care, the study of moral sensitivity can help to create and consolidate work teams and to generate environments and strategies that favor an ethical climate, which contribute to maintaining and increasing the quality of care, (17) which can be affected by high levels of work stress and anxiety(24) because a high level of moral sensitivity could help nurses to confront the situation and defend their autonomy and moral integrity.

The degree of moral sensitivity determined by the dimension of *Values* in the participating nurses corresponds to 90%, a higher finding than that published by Campillo,(17) who reported 75.7%. Most of the participants consider that moral sensitivity in caring for the terminally ill is based principally on values because, for them, being morally sensitive implies having professional availability, establishing interpersonal relationships of trust, supporting the patient -above all in times of suffering-, being attentive to their needs and, even, appropriate communication with the physician. A similar finding was obtained in another study(17) in which the items with higher score over moral sensitivity were, in their order: “in my work environment, I consider it fundamental to show the patient an attitude of support”, “I believe that as a nurse, I must help patients to express their concerns to the physician”, “in my work environment, I consider it fundamental to establish a relationship of trust with the patient”, “for me, it is important - as a nurse - to express to the patient my availability as a professional”, “I feel I must assure patients that, as a nurse, I will be available to support them in times of suffering”, and “being attentive to patients’ expressions helps me to perceive their needs”. 

According to that expressed by Campillo,(17) the moral sensitivity of nursing professionals in any care activity is evidenced through values of responsibility, honesty, and trust; this aspect coincides with the ideas by Watson,(25) who expresses that care must be based on a system of humanist-altruist values that grant nurses the possibility of giving and receiving, appreciating the whole diversity of life and their individuality. Elliott,(26) in turn, in a systematic review, found that respect, altruism, honesty, responsibility, compassion, commitment, trust, and social justice are values that give identity to the nursing professional. For the participants, particularity and individuality are significant in caring for the terminally ill, given that the majority considered it important to recognize that each being is unique and should not be subordinated to a medical diagnosis. This contrasts with that found by Campillo,(17) who reports a lower score in the items “I have a special interest in helping patients realize that each being is unique” and “it bothers me to hear patients being referred to by their diagnosis”. 

When caring for the terminally ill, nurses confront the vulnerability, needs of the patient and the different ethical dilemmas involving each particular situation; perceiving these and providing care responses evidences their degree of moral sensitivity. 

This study found an average of 38.7, which corresponds to 70.4% of moral sensitivity -similar data to the 70.7% found in a study(17) about care responses. In research like this, most of the nurses agreed that in front of terminal patients it is necessary to show a calm attitude, provide comfort to the sick, and help them identify their strengths and capabilities. Likewise, they harmonize in that related aspects on being morally sensitive in nursing means having attitudes, like patience, active listening, observation, openness, and transparency, seeking comfort, tact, comprehension and promotion of self-esteem;(23) a peaceful end-of-life experience is achieved when nurses provide wellbeing by preventing, controlling, and relieving physical discomfort, facilitating rest, satisfaction, and preventing complications in patients.(9) At the end of life, it is necessary to seek a calm environment and an atmosphere of serenity and peace; for such, nurses will strengthen the family bond and that of friends, will meet the desires and preferences, and the needs for communication of patients and their families; likewise, nurses must support patients in their construction of sense, that is, help them to resignify death, what is left of life and plan it near death.(9)

Over half the nurses who participated in this study believe that sometimes they impose their values on the patient, it is difficult for them to identify concerns with respect to religious expressions (candles, fasting, or feeding practices) and accept certain decisions by the patients. In the study by Campillo,(17) nurses expressed difficulty in accepting and respecting expressions regarding the beliefs and values of the people. These care responses mediated by particular values and beliefs indicate that nurses would be upon an ethical conflict and of values with respect to those professed by the patient, given that many of the decisions made regarding care do not always agree with the patient’s system of values. 

## Conclusion.

The nurses participating in this study have high moral sensitivity to care for patients in terminal state. The dimension of values evidences the respect for the beliefs and values of the people and for their capacity to decide. The results obtained in the dimension of care responses show that the study participants have difficulties in accepting the diversity of expressions emerging while caring for patients. Within the setting of nursing care, this study of moral sensitivity indicates that strategies must be formulated to favor an ethical climate and which contribute to improve the quality of care, which can be affected by high levels of work stress and anxiety, given that a high level of moral sensitivity could help nurses to face the situation and defend their autonomy and moral integrity.
